# 

**Published:** 1874-10

**Authors:** 


### CONTENTS:

### ORIGINAL COMMUNICATIONS.

ARTICLE I.—Ætiology of Dental Caries. By Dr. A. W. Harlan........ 241

ARTICLE II.—The Changes in the Shapes of Teeth that are Necesary or Proper for the Treatment of Decay. Dr. Edmund Noyes........ 250

ARTICLE III.—The Part which Vital Action plays in the History of Dental Caries. By Dr. H. S. Chase........ 256

### SELECTED ARTICLES.

ARTICLE IV—The Action of Different Medicines Used in Dental Practice. By Dr. Jno. Murray........ 261
ARTICLE V.—On Amalgams. By Dr. Amos Kirby........ 270
ARTICLE VI—Annual Meeting of the American Dental Association........ 276
ARTICLE VII.—A Safe and Simple Method for Self-administration of Chloroform. By W. W. Lane, M. D........ 278
ARTICLE VIII.—Carvacrol. By Dr. E. A. Bogue........ 279

### EDITORIAL, ETC.

Vulcanite Litigation........ 280
How to Use Plastic Fillings........ 282

### MONTHLY SUMMARY.

School Diseases........ 283
Camphorated Phenol........ 284
Anæsthetization During Sleep........ 285
Influence of Fear in the Production of Disease........ 286
The Position of Woman........ 286
The Hours of Maximum Mortality in Acute and Chronic Diseases........ 287
Chlorate of Potash in Cancer........ 287
The Champion Chloroform Tippler........ 287

